# Priming Astrocytes With HIV-Induced Reactive Oxygen Species Enhances Their *Trypanosoma cruzi* Infection

**DOI:** 10.3389/fmicb.2020.563320

**Published:** 2020-10-19

**Authors:** Javier Urquiza, Cintia Cevallos, María Mercedes Elizalde, M. Victoria Delpino, Jorge Quarleri

**Affiliations:** ^1^Instituto de Investigaciones Biomédicas en Retrovirus y Sida (INBIRS), Facultad de Medicina, Universidad de Buenos Aires (UBA), Buenos Aires, Argentina; ^2^Consejo Nacional de Investigaciones Científicas y Técnicas (CONICET), Buenos Aires, Argentina; ^3^Instituto de Inmunología, Genética y Metabolismo (INIGEM), Facultad de Farmacia y Bioquímica, Universidad de Buenos Aires, Buenos Aires, Argentina

**Keywords:** astrocytes, *Trypanosoma cruzi*, human immunodeficiency virus, reactive oxygen species, extracellular vesicles

## Abstract

**Introduction**: *Trypanosoma cruzi* is an intracellular protozoa and etiological agent that causes Chagas disease. Its presence among the immunocompromised HIV-infected individuals is relevant worldwide because of its impact on the central nervous system (CNS) causing severe meningoencephalitis. The HIV infection of astrocytes – the most abundant cells in the brain, where the parasite can also be hosted – being able to modify reactive oxygen species (ROS) could influence the parasite growth. In such interaction, extracellular vesicles (EVs) shed from trypomastigotes may alter the surrounding environment including its pro-oxidant status.

**Methods**: We evaluated the interplay between both pathogens in human astrocytes and its consequences on the host cell pro-oxidant condition self-propitiated by the parasite – using its EVs – or by HIV infection. For this goal, we challenged cultured human primary astrocytes with both pathogens and the efficiency of infection and multiplication were measured by microscopy and flow cytometry and parasite DNA quantification. Mitochondrial and cellular ROS levels were measured by flow cytometry in the presence or not of scavengers with a concomitant evaluation of the cellular apoptosis level.

**Results**: We observed that increased mitochondrial and cellular ROS production boosted significantly *T. cruzi* infection and multiplication in astrocytes. Such oxidative condition was promoted by free trypomastigotes-derived EVs as well as by HIV infection.

**Conclusions**: The pathogenesis of the HIV-*T. cruzi* coinfection in astrocytes leads to an oxidative misbalance as a key mechanism, which exacerbates ROS generation and promotes positive feedback to parasite growth in the CNS.

## Introduction

The intracellular protozoan *Trypanosoma cruzi* (*T. cruzi*) causes Chagas disease, which is one of the most important current neglected diseases in the Americas and increasingly widespread worldwide. It is transmitted to humans by *Triatoma infestans* as vector. Current estimations from the World Health Organization (WHO) denote that 8 million people are infected and other 25 million people are vulnerable of acquiring *T. cruzi* infection worldwide (Perez-Molina and Molina, 2018). Over the last decades of the twentieth century, chronically infected individuals with severe immunosuppression, such as transplant recipients and people living with HIV/AIDS (PLWHA), were at risk of severe forms of reactivation, such as Chagasic meningoencephalitis (Lattes and Lasala, 2014). This condition can arise at chronic stage of infection in immunocompromised hosts including PLWHA (Bern et al., 2011), and it is characterized by brain nodular lesions usually called chagomas involving macrophages, neutrophils, microglia, astrocytes, and perivascular lymphocytic infiltrate in various foci along the central nervous system (CNS; Gomez and Banaei, 2018). Astrocytes are the most abundant brain cells and have multiple functions including maintenance of an adequate environment for neurons by providing metabolic support and modulating synaptic transmission. Moreover, they promote myelination activity of oligodendrocytes and nervous systems repair (Sofroniew and Vinters, 2010; Kiray et al., 2016).

*T. cruzi* life cycle comprises the trypomastigote as infective bloodstream-form able to infect different nucleated cells. Cell infection is a complex process and includes the parasite-cell uptake, an endocytic process with modification of cellular cytoskeleton, and the growth of a parasitophorous vacuole that encloses trypomastigotes. These trypomastigotes escape into the cytoplasm to be transformed in the amastigote form, able to multiply intracellularly by binary division. Intracellular amastigotes sustain metabolic dependence on cellular energy, nucleotide and fatty acid/glucose metabolites, and survival signaling suggesting a role of host cell metabolism in regulating their intracellular growth (Caradonna et al., 2013). Finally, when the host cell is overwhelmed, amastigotes differentiate into trypomastigotes, host cell is lysed, and infective parasites are released to the bloodstream and eventually could access to the CNS.

Extensive clinical, cerebrospinal fluid (CSF), neuroimaging, and neuropathological data support persistent HIV infection in the CNS (Valcour et al., 2011). We and others have demonstrated that, like other CNS cells, astrocytes can host several infectious agents, including HIV and *T. cruzi* (Blanchet et al., 2010; Vargas-Zambrano et al., 2013; Urquiza et al., 2017) and are regarded as important performers in Chagasic meningoencephalitis development. HIV can infect the brain and impair CNS function. HIV does not directly infect neurons but causes neuronal damage by affecting microglia and other cells in the CNS including astrocytes, causing primary CNS injury (Ellis et al., 2009). This evidence indicated that the cellular response at the local level is altered during HIV infection and the responses to a secondary infection such as *T. cruzi* could be altered favoring the parasite survival and multiplication. Moreover, HIV-infected astrocytes also alter the blood-brain barrier (BBB) permeability due to endothelial cell apoptosis through the disruption of gap junction (Eugenin et al., 2012). This bystander BBB toxicity mediated by HIV-infected astrocytes contributes to understanding not only how the CNS damage caused by HIV has spread but also the increased entry of other pathogens to the CNS. Therefore, Chagas disease may be reactivated in the CNS due to HIV-induced immunosuppression.

As an obligatory intracellular parasite and due to the variety of cellular host environments, *T. cruzi* faces several sources of reactive oxygen species (ROS) including ROS produced by its metabolism and ROS generated by the host’s immune responses. Paradoxically, besides the role of ROS in pathogen elimination during the oxidative burst, increasing evidence suggests that ROS production is beneficial to *T. cruzi* infection (Gupta et al., 2009; Paiva et al., 2012, 2018; Goes et al., 2016). This paradoxical role of ROS could be a new target for anti-parasite therapy improvement (Providello et al., 2018). During the cellular invasion, trypomastigotes can activate regulatory pathways using a large number of molecules (Bayer-Santos et al., 2013) carried by extracellular vesicles (EVs), which are small membrane-bound vesicles able to transport secretome components of *T. cruzi* (Retana Moreira et al., 2019). Nevertheless, it remains unclear whether EVs released from parasitized cells and/or by trypomastigotes play any role on *T. cruzi* infection and multiplication by altering the cell host pro-oxidant status.

In the present work, we investigated how the pro-oxidant condition self-promoted by the parasite using its own trypomastigotes derived-EVs or by HIV infection is able to influence *T. cruzi* infection and multiplication in human astrocytes.

## Materials and Methods

### Astrocyte Cultures and *T. cruzi* Strain

Normal human astrocytes (NHA; Lonza®, Pharma&Biotech-Bioscience Solutions) were used. NHA were seeded and grown in AGM™ Bullet Kit™ medium (Lonza®) at 37°C with 5% CO_2_.

*T. cruzi* K98 clone belonging to the discrete typing unit I (DTU I) was used. The clone K98 of the parasite was genetically modified to express the enhanced green fluorescent protein (eGFP). The parasite depicts a homogeneous pattern of GFP fluorescence that allowed to measure both the infection and the multiplication rate in astrocytes. *T. cruzi* was preserved in monolayers of Vero cells (ECACC 84112001). After being released from infected cells, trypomastigotes were collected from supernatants by low-speed centrifugation (500 rpm, 10 min) in order to remove any contaminating cell debris and then counted in a Neubauer chamber.

### HIV env-Pseudotyped Production, Titration, and Infection Efficiency Measurement

The pNL4-3 is a full-length infectious molecular clone of HIV that utilizes CXCR4 as a co-receptor. The NL4-3-GFP and pNL4-3-DsRed are two molecular clones that contain the enhanced version of the green fluorescent protein gene (eGFP) or the *Discosoma* sp. red fluorescent protein (DsRed, Clontech), respectively. These viral clones were used alternatively according to fluorochromes requirements for microscopy or flow cytometry evaluation. High-titer stocks of HIV env-pseudotyped with vesicular stomatitis virus envelope glycoprotein (VSV-G) were obtained by cotransfecting 293T cells with a VSV-G expression plasmid (Clontech) at HIV/VSV-G plasmid ratio of 10:1. The concentration of virus stocks was adjusted to a titer of 100 ng/μl of p24 (HIV capsid protein). Viral infection efficiency was monitored as a function of time using three different approaches: (1) HIV p24 capsid protein in cell culture supernatants (p24 ELISA kit, INNOTEST HIV Antigen mAb), (2) HIV p24 intracellular expression using the KC57 monoclonal antibody labeled with fluorescein isothiocyanate against p24 (p24-FITC) protein (Beckman Coulter, United States; Chassagne et al., 1986; Darden et al., 2000), and (3) HIV gene expression by GFP or DsRed measurement by flow cytometry (FACSCanto II flow cytometer, BD Biosciences, San Jose, CA, United States). Additionally, the identification of productively infected cells for each pathogen, eventual cohabitation of both and neighbor-uninfected cells in a heterogeneous population was measured using flow cytometry analysis of cell fluorescence, by, allows. The measurement of fluorochrome expression sensitivity and performance were checked before data acquisition using Cytometer Setup & Tracking beads (BD Biosciences, San Jose, CA, United States). All experiments were performed in a BSL-3 laboratory at the INBIRS.

### HIV and *T. cruzi* Infection of Astrocytes

For infection, human astrocytes were seeded in 48-well culture plates at 3 × 10^4^cells/well. The HIV infection was carried put using an inoculum of 8 μg/ml (measured as p24 antigen), while *T. cruzi* infection with trypomastigotes used a parasite:cell ratio of 5:1. Hence, we used 1.5 × 10^5^ trypomastigotes/well. In these conditions, the release of trypomastigotes began 5-day post-exposure (dpe) from *T. cruzi*-infected astrocytes. To ensure the absence of free HIV particles before inoculating the parasite, astrocytes were washed five times with fresh DMEM.

### Evaluation of the *T. cruzi* Infection and Multiplication

The level of infection and multiplication of *T. cruzi* in astrocytes were measured by flow cytometry. For debris exclusion, astrocytes were gated based on side scatter and forward scatter. Quantification of astrocytes with specific fluorescence as well as the mean fluorescence intensity (MFI) in those cells was indicative of infection and multiplication efficiency, respectively. We have collected, stored, and analyzed the data from 5 × 10^4^ cells using Flow Jo X software for Windows 7.0.

*T. cruzi* infection was also evaluated by fluorescence microscopy. The presence of intracellular amastigotes was evaluated using rabbit polyclonal anti-*T. cruzi* antibody (kindly donated by Dr. Federico Penas, INBIRS, Universidad de Buenos Aires, Argentina). Then, a commercial goat anti-rabbit IgG (H + L) secondary antibody, Alexa Fluor 647 (Invitrogen, Thermo Fisher) was used. The coverslips mounted with DAPI Fluoromount-G (Southern Biotech) were studied in a Nikon Eclipse Ti-S L100 fluorescence microscope using a Plan Apochromat 60 × 1.42 NA oil immersion objective. Images were analyzed using the NIS-Element software.

Finally, a standardized real-time PCR-based method was carried out to quantify intracellular *T. cruzi* DNA (Schijman et al., 2011). For this purpose, after the solvent-based DNA extraction, a Sybr-Green-based real-time PCR targeted to parasite satellite DNA (Sat-DNA) using specific primers TCZ-F (5'-GCTCTTGCCCACAMGGGTGC-3') and TCZ-R (5'-CCAAGCAGCGGATAGTTCAGG-3') was carried out.

### Astrocyte Apoptosis Measurement by Flow Cytometry

Using flow cytometry, the percentage of early apoptotic cells was defined based on the plasma membrane permeability and phosphatidylserine cell translocation measurement. For this purpose, dual staining was done with PE or APC-conjugated Annexin-V and 7-amino-actinomycin D (7AAD) using the Annexin-V/7AAD apoptosis detection kit (BD Biosciences).

### Isolation, Quantification, and Characterization of Extracellular Vesicles

Free trypomastigotes of *T. cruzi* and human astrocytes were washed with serum-free DMEM. Subsequently, secretion equivalents were obtained from 10^8^ parasites and 10^7^ astrocytes as was previously described with modifications (Caeiro et al., 2018). For this purpose, such parasite and human cells were incubated in DMEM 10% Gibco Exosome-depleted fetal bovine serum (FBS) at 37°C for 6 h in a humidified atmosphere with 5% CO_2_. The products secreted by trypomastigotes and astrocytes were isolated by centrifugation and filtration as follows: (a) 3,000 *g* for 10 min at 4°C to remove the trypomastigotes and apoptotic bodies followed by (b) ultracentrifugation at 100,000 *g* at 4°C to generate the pellet of EVs followed by the second ultracentrifugation with PBS to remove impurities, and finally, (c) the cell-free supernatant was filtered with 0.45 μm syringe filters. The supernatant was wasted, and the exosome pellet was resuspended in 200 μl–1 ml PBS for scanning electron microscopy (SEM) inspection. All ultracentrifugation stages were performed in a 70Ti fixed angle rotor in an Optima XL 100 k ultracentrifuge (Beckman Coulter). For each pellet, protein quantification was spectrophotometrically performed using the Bio-Rad Protein Assay reagent and bovine serum albumin (BSA; 1 mg/ml) as standard.

To assess the biochemical characteristics of EVs by Western Blot, 40 μg of EVs isolated from 100 × 10^6^ of trypomastigotes obtained in Vero cells, and 10 × 10^6^ astrocytes or their equivalent of soluble vesicle secretion (EVs) were electrophoresed in 12% denaturing polyacrylamide gels and transferred to PVDF membranes by standard methodologies. The appropriate transfer was tested by reversible membrane staining with Ponceau Red (5% w/v) in 1% acetic acid (v/v). The membrane was blocked with TBS-3% non-fat milk for 1 h, washed with TBS-0.05% Tween and incubated with primary antibodies. After several washes, membranes were incubated with the secondary antibody horseradish peroxidase-conjugated goat anti-mouse IgG (1∶4000) and developed with the Supersignal® West Pico Chemiluminescent Substrate (Pierce) according to the manufacturer’s instructions. The revealed images were acquired by the MultiDoc-It™ Imaging System. Western blotting of EVs of *T. cruzi* was carried out using primary mouse antibodies anti-TcTASV-C (Trypomastigote Alanine, Serine and Valine protein from *T. cruzi*; 1/400) and anti-SAPA (Shed Acute-Phase Antigen, a trans-sialidase repetitive domain) kindly provided by Dr. Valeria Tekiel (IIB-INTECH, UNSAM-CONICET, Argentina). Mouse anti-CD9 (clone M-L13; BD Bioscience) and anti-CD63 antibodies (clone H5C6; BD Bioscience) were used for EVs derived from astrocytes. As negative controls, two cytoplasmic proteins were detected: rabbit polyclonal anti-calnexin (1/500; Abcam) and rabbit polyclonal anti-SR62 (1/1000), which must be absent in pure EVs of astrocytes and trypomastigotes, respectively.

For SEM, pellets containing EVs isolated from healthy astrocytes, parasitized cells, and trypomastigotes were vortexed and resuspended in 200 μl–1 ml PBS. EVs were fixed in 2% SEM-quality paraformaldehyde aqueous solution and then diluted in distilled water and added in 1–5 μl vesicle mixtures to clean silicon chips. Samples were mounted on a SEM stage by carbon paste. SEM (Hitachi S-4700) was performed under low beam energies (5.0–10.0 kV). Analysis of EVs sizes was done using the SEM images *via* ImageJ (Wayne Rasband, NIH, United States).

Astrocyte (3 × 10^4^ cells/well) stimulation with free *T. cruzi*-derived EVs was performed using 6 μg/well of protein. Such amount of protein was obtained from EVs extracted from 1.5 × 10^5^ trypomastigotes. Equal protein concentration was used when challenges were performed with EVs from the other two sources (Tc-infected astrocytes and uninfected astrocytes). The EV-treated astrocytes were exposed to *T. cruzi* at three distinguishable conditions such as 24 h before, simultaneous, or 24 h after EVs exposure.

### Detection and Scavenging of Cellular Reactive Oxygen Species Generation

Production of cellular and mitochondrial ROS was evaluated using DCFDA and MitoSOX assays, respectively as follows:

a. Cellular ROS production (including hydroxyl, peroxyl, and another ROS) were measured using a DCFDA assay kit (Abcam, United States) according to the manufacturer’s protocol. Following a specific timeline after infection, cells were washed twice with PBS, incubated with 25 μM DCFDA in the essential medium at 37°C for 45 min, and evaluated by flow cytometry.
b. Cellular mitochondrial ROS measurement by flow cytometry was made by staining with MitoSOX™ (Thermo Fisher Scientific, United States) following the manufacturer’s instructions. Briefly, at the predetermined time points after infection, cells were washed twice with PBS, incubated with 5 μM MitoSOX at the corresponding temperature and time for each reagent, and then evaluated by flow cytometry.

Also, astrocytes were pre-treated for 18 h with the antioxidant ascorbic acid (AA) or with MitoTEMPO® (MT; Sigma-Aldrich), a well-known mitochondria-specific superoxide scavenger, to validate the *in vitro* model and to demonstrate ROS functionality. AA is a non-enzymatic antioxidant, contributing to ROS-scavenging. A stock solution of 100 mM (Sigma) was prepared fresh in a modified Krebs buffer and then diluted in cell culture media until 1 mM final concentration. MT was used in a final concentration of 10 μM.

Tert-Butyl hydroperoxide (TBH) is an organic peroxide used as ROS inducer. Here, three serial dilutions were prepared (100, 50, and 10 μM) from a 70% aqueous solution (Sigma-Aldrich). Astrocytes treated with increased concentrations of TBH (1 h, 37°C) were used as positive controls for ROS generation.

### Statistical Analysis

Where applicable, statistical analysis was performed. Multiple comparisons between all pairs of groups were made with Tukey’s test, and those against two groups were made with Mann-Whitney U test. Graphical and statistical analyses were performed with GraphPad Prism 5.0 software.

Each experiment was performed in triplicates with different culture preparations on five independent occasions. Data were represented as mean ± SD measured in triplicate from three individual experiments. A *p* < 0.05 is represented as ^*^, *p* < 0.01 as ^**^, and *p* < 0.001 as ^***^. *p* < 0.05 was the minimum level for accepting a statistically significant difference between groups.

## Results

### *T. cruzi* Induces ROS Upregulation in Astrocytes Favoring Its Infection and Multiplication

**Figure 1:**
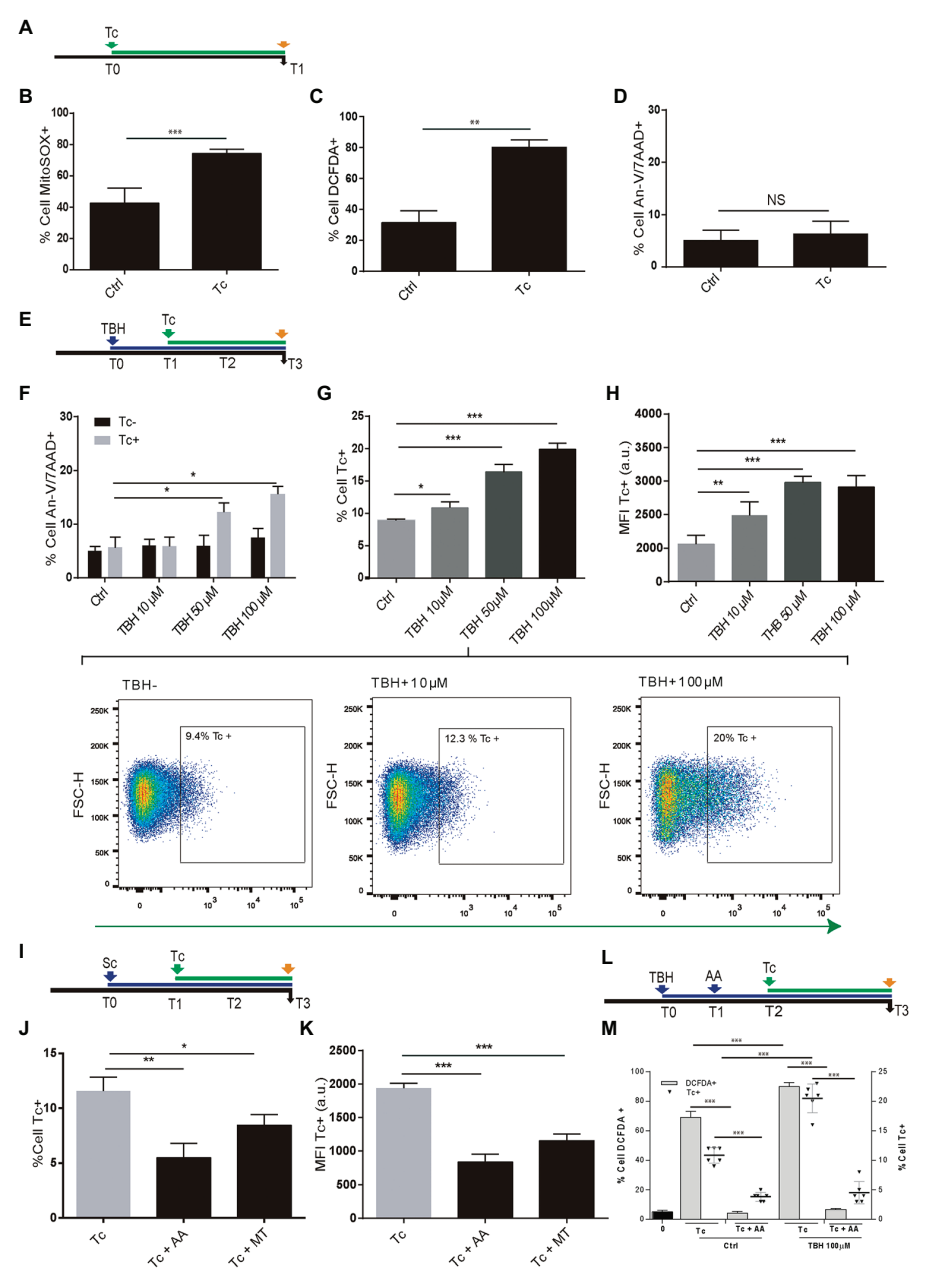
Effect of *Trypanosoma cruzi* infection on astrocyte ROS level and its consequences on parasite infection and multiplication. **(A)** Timeline of the Tc infection of cultured human astrocytes. Parameters were measured at T1 (orange arrow, 24 h after Tc exposure). **(B)** mROS level was measured by flow cytometry using MitoSOX in uninfected astrocytes – Ctrl – and Tc-infected astrocytes. **(C)** cROS level was measured by flow cytometry using DCFDA (assumed to be proportional to the concentration of hydrogen peroxide) in uninfected astrocytes – Ctrl – and Tc-infected astrocytes. **(D)** Cell death levels (positive staining for annexin-V and 7-AAD) in uninfected (Ctrl) and Tc-infected astrocytes analyzed by flow cytometry. **(E)** Timeline of the exposure of cultured astrocytes to tert-Butyl hydroperoxide (TBH; blue arrow) followed by Tc infection (green arrow, 1 h after TBH-exposure). Parameters were measured at T3 (orange arrow, 48 h after Tc exposure). **(F)** Cell death levels by flow cytometry (positive staining for annexin-V and 7-AAD) in unexposed (Ctrl) and TBH-exposed cells (at increasing concentrations as indicated), in non-infected (black columns) and Tc-infected (gray columns) astrocytes. **(G)** Tc-infection rate analysis in unexposed (Ctrl) and TBH-exposed cells (at increasing concentrations as indicated). Three representative dot plots representation are shown. For each diagram, a square depicted the percentage of Tc-infected cells (GFP-positive). **(H)** Tc-multiplication quantification by flow cytometry as mean fluorescence intensity (MFI, expressed in arbitrary units) in unexposed (Ctrl) and TBH-exposed cells (at increasing concentrations as indicated). **(I)** Timeline of the exposure of cultured astrocytes to ROS scavengers (MT: MitoTEMPO; AA: ascorbic acid; blue arrow) followed by Tc infection (green arrow, 18 h after scavengers exposure). Parameters were measured at T3 (orange arrow, 48 h after Tc exposure). **(J)** Tc-infection rate analysis in non-scavenged and scavenged (with MT, or AA) *T. cruzi*-infected cells. **(K)** Tc-multiplication quantification by flow cytometry as MFI in non-scavenged and scavenged (with MT, or AA) *T. cruzi*-infected cells. **(L)** Timeline of the exposure of cultured astrocytes to pro-oxidant condition (TBH, 100 μM during 1 h), ROS scavenging (AA: ascorbic acid), and Tc infection (green arrow, 18 h after AA exposure). Parameters were measured at T3 (orange arrow, 48 h after Tc exposure). **(M)** Astrocyte ROS level (% cell DCFDA+) and Tc infection rate (% cell Tc+) analysis in control and TBH-treated astrocytes, with and without scavenging (AA). Graphics are showing values obtained from three independent experiments. Data are given as the mean ± SD. NS (not significant), ^*^p < 0.05, ^**^p < 0.01, and ^***^p < 0.001.

During *T. cruzi* infection, the macrophages respiratory burst produces ROS. However, the parasite has an antioxidant machinery to deal with the oxidative burst. Moreover, such pro-oxidant environment fuels the *T. cruzi* infection (Gupta et al., 2009; Paiva et al., 2012, 2018; Goes et al., 2016). Here, we have examined whether ROS production occurs during the parasite infection of astrocytes and the impact on its multiplication. For this goal, we performed experiments using MitoSOX and DCFDA, useful for measurement of mitochondrial ROS (mROS) and cellular ROS (cROS) activity by flow cytometry, respectively. Twenty-four hours after *T. cruzi* (trypomastigotes) challenge (Figure 1A), significantly higher mitochondrial and cellular ROS positive cells were measured in infected astrocytes to control cells (Figures 1B,C). However, these higher levels of ROS observed after *T. cruzi* exposure did not modify the level of programed cell death in comparison with control astrocytes (Figure 1D).

Furthermore, to evaluate the ROS impact on *T. cruzi* infection and multiplication, astrocytes were treated with 10, 50, and 100 μM TBH for 1 h before being exposed to trypomastigotes (Figure 1E). The level of cell death was evaluated after 48 h in both conditions: control cells (only exposed to TBH) and TBH/*T. cruzi*-infected cells.

In uninfected cells, the level of cell death after TBH treatment did not depict any significant differences according to growing (10, 50, and 100 μM) pro-oxidant concentration respect to untreated control. But the level of cell death on astrocytes was significantly higher in *T. cruzi*-infected cells in the presence of 50 and 100 μM of TBH as shown in Figure 1F.

The impact of TBH-induced pro-oxidant level on *T. cruzi* infection and multiplication was measured 48 h post-parasite exposure. The infection rate of astrocytes by *T. cruzi* was directly and dose-dependently influenced by the level of ROS. Cells exposed to growing doses of TBH (10, 50, and 100 μM) showed significantly higher levels of infection compared to unexposed controls (Figure 1G). Likewise, a significantly higher level of amastigote multiplication was found when astrocytes were under pro-oxidant condition maintaining a dose-dependent response according to increased TBH level in comparison with unexposed controls (Figure 1H).

The incumbency of the pro-oxidant environment on *T. cruzi* infection and multiplication was also evaluated by diminishing the ROS activity using mitochondrial and cytoplasmic scavengers (MT and AA, respectively) before parasite infection (Figure 1I). When the ROS level was depleted using scavengers, significantly lower infection and multiplication were observed (Figures 1J,K). Finally, the influence of both, the TBH-pro-oxidant environment (using 100 μM) and the ROS scavenging (using AA) on both *T. cruzi* infection rate and ROS level (measured using DCFDA) were simultaneously measured. Thus, the parasite infection rate and the ROS level among astrocytes previously exposed to TBH were significantly higher than control (non-TBH exposed) cells. Although, when ROS scavenging with AA was applied on astrocytes (initially exposed or not to TBH) after their parasite challenge both, Tc infection rate and ROS level appeared significantly reduced (Figures 1L,M).

Taken together, our results indicate that astrocytes primed by an oxidative environment or by their oxidative stress – with cellular and mitochondrial ROS production – enhance their *T. cruzi* infection and multiplication.

### Extracellular Vesicles Enhance *T. cruzi* Infection by Inducing ROS Upregulation

**Figure 2:**
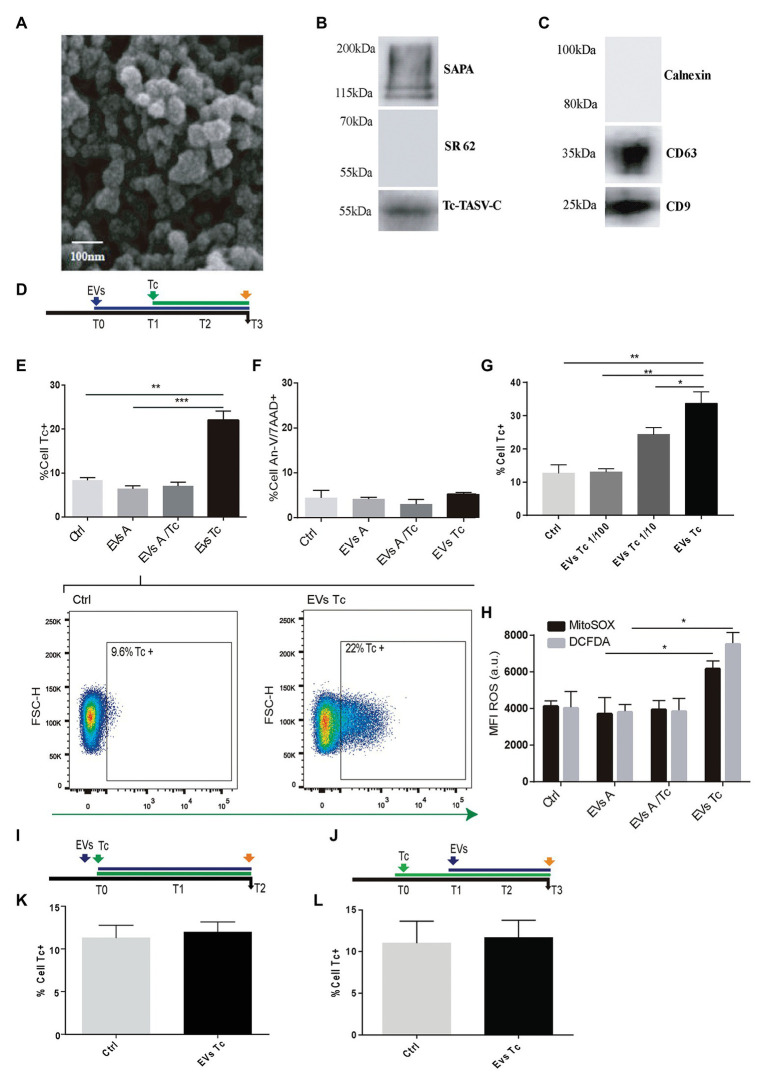
Effect of trypomastigotes-derived extracellular vesicles (EVs) in astrocytes ROS concentration and *Trypanosoma cruzi* infection. **(A)** Scanning electron microscopy (SEM) showing clusters of trypomastigotes-derived EVs formed by ultracentrifugation. The bar size is indicated. **(B)** Biochemical characterization of EVs purified from trypomastigotes by western blot to verify the presence of SAPA (shed acute-phase antigen) and Tc-TASV-C antigens and the absence of SR62 (*T. cruzi* cytoplasmic antigen). **(C)** Biochemical characterization of EVs purified from astrocytes. The presence of CD63, CD9, and absence of calnexin was determined by western blot from astrocyte-derived EVs. **(D)** Timeline of the EVs exposure (blue arrow) in cultured astrocytes followed by Tc infection (green arrow, 24 h after EVs exposure). Parameters were measured at T3 (orange arrow, 48 h after Tc exposure). **(E)** Tc-infection rate measured as GFP+ cells analysis in EVs non-exposed astrocytes followed by Tc infection (Tc), and EVs-exposed cells [EVs from normal astrocytes (EVs A), Tc-infected astrocytes (EVs A/Tc), and EVs from free trypomastigotes (EVs Tc)]. Two representative dot plots obtained by flow cytometry showing EVs non-exposed astrocytes (Ctrl, left panel) and EVs (from trypomastigotes) exposed-astrocytes (EVs-Tc, right panel). In each diagram, a square depicted the percentage of Tc-infected cells (GFP-positive). **(F)** Astrocyte death levels (measured as positive staining for annexin-V and 7-AAD) in EVs non-exposed astrocytes followed by Tc infection (Ctrl), and EVs-exposed cells [with EVs obtained from cell sources defined in item **(E)**]. **(G)** Tc-infection rate measured as GFP+ cells analysis in EVs from free trypomastigotes (EVs Tc) at dilutions 1/100 (0.06 μg/well), 1/10 (0.6 μg/well), and pure (6 μ/well). **(H)** Cellular and mitochondrial ROS level (using DCFDA – represented in gray columns and MitoTEMPO – represented in black columns, respectively) as MFI, in astrocytes (exposed to EVs from sources described in **E**). **(I)** Timeline of the EVs exposure (blue arrow) in cultured astrocytes simultaneously infected with Tc (green arrow). Parameters were measured at T2 (orange arrow, 48 h after Tc + EVs exposure). **(J)** Timeline of the cultured astrocytes infected with Tc (green arrow) and stimulated with EVs (blue arrow, 24 h after Tc exposure). Parameters were measured at T3 (orange arrow, 48 h after EVs exposure). **(K)** Tc-infection rate measured as GFP+ cells analysis in EVs non-exposed astrocytes but Tc-infected (Ctrl), and EVs from free trypomastigotes-exposed cells simultaneously with Tc infection. **(L)** Tc-infection rate measured as GFP+ cells in EVs non-exposed astrocytes but Tc-infected (Ctrl), and EVs from free trypomastigotes-exposed cells after infection with Tc. Graphics are showing values obtained from three independent experiments. Data are given as the mean ± SD Significant differences are indicated by ^*^p < 0.05, ^**^p < 0.01, and ^***^p < 0.001, respectively.

Considering their role in parasitism increment (Retana Moreira et al., 2019), the capability of EVs released from *T. cruzi* to alter the host-cell oxidant environment was evaluated. EVs were obtained after incubation of 10 × 10^7^ parasite tissue-culture cell-derived trypomastigotes (K98 strain). These EVs were characterized by Scanning Electronic Microscopy (SEM), and the presence of *T. cruzi* trypomastigotes surface molecules (SAPA and Tc-TASV-C antigens) was assessed by western blotting (Figures 2A,B). Besides, EVs obtained from 10 × 10^6^ astrocytes parasitized by *T. cruzi* amastigotes as well as from non-infected astrocytes. These EVs were biochemically characterized by detecting cell-derived EVs canonical proteins CD9 and CD63 (Figure 2C).

When astrocytes were exposed to EVs released by free trypomastigotes (but not from EVs released from Tc-infected cells and EVs from uninfected cells) during 24 h, a significant increase in the ROS level was observed respect to untreated cells. The influence of this EVs-induced oxidant environment on the *T. cruzi* infection rate was evaluated following an analogous timeline than previous ones (Figure 2D). In this condition, the infection rate was increased significantly without infringing significant changes in cell viability (Figures 2E,F). This EVs-mediated parasitism augmentation phenomenon showed a dose-dependence since it was significantly reduced when exposures were carried out with 1/10 and 1/100 dilutions of EVs (0.6 and 0.06 μg/well, respectively; Figure 2G). The ROS (cellular and mitochondrial) level in astrocytes exposed to trypomastigotes-derived EVs was significantly higher than control cells. In contrast, such ROS levels in astrocytes did not experience any change after exposure to EVs released from uninfected and parasitized astrocytes (Figure 2H).

To determine whether the EVs-induced *T. cruzi* infection enhancement keeps a time-dependence, we have evaluated two additional conditions as follows: (i) simultaneous challenge of astrocytes with trypomastigotes-derived EVs and *T. cruzi* (Figure 2I) and (ii) astrocytes exposure to free trypomastigotes-derived EVs after their *T. cruzi* exposure (Figure 2J). In both conditions, the *T. cruzi* infection rate did not differ significantly from control cells (Figures 2K,L, respectively). Likewise, the ROS level was similar in these two conditions (data not shown).

Taken together, these results demonstrate that EVs from free *T. cruzi* trypomastigotes prime astrocytes by raising their ROS level, thus enhancing the parasite infection and multiplication.

### *T. cruzi* Infection and Multiplication in Astrocytes Is Enhanced by HIV Infection

**Figure 3:**
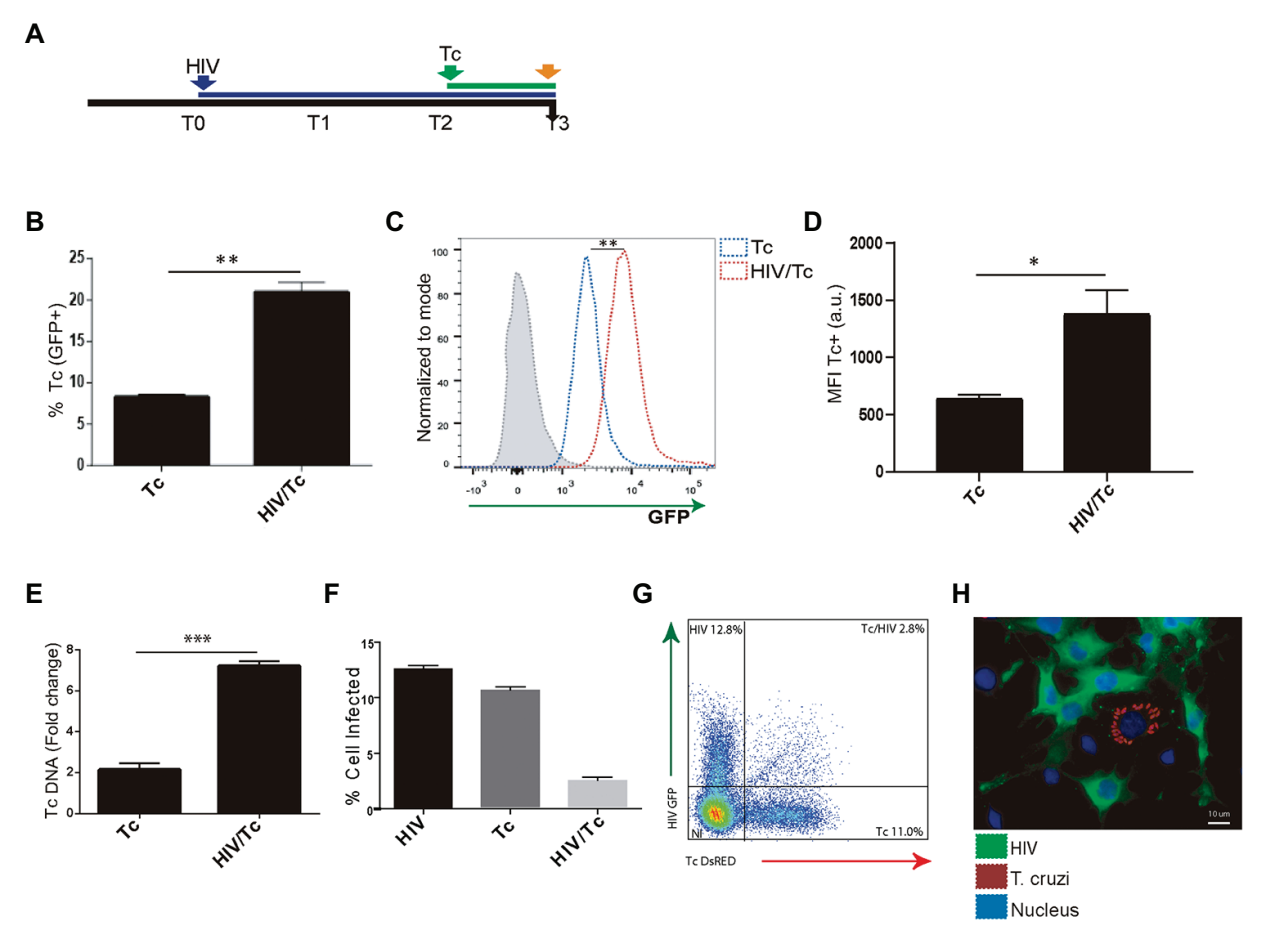
Effect of HIV infection of astrocytes on *Trypanosoma cruzi* infection and multiplication. **(A)** Timeline of the HIV (T0, blue arrow) and Tc (T2 green arrow, after 48 h of HIV exposure) infection of cultured human astrocytes. Parameters were measured at T3 (orange arrow, 24 h after Tc exposure). **(B)** Effect of HIV pre-infection of astrocytes on the Tc-infection rate measured by flow cytometry analysis as percentages of GFP+ cells. **(C)** Histogram example of the quantitative analysis of Tc-GFP positive cells proportion and MFI measured by flow cytometry. The *x*-axis represents the fluorescent signal intensity, and *y*-axis the normalized cell number expressed as a percentage of the maximum. **(D)** Relationship between the geometric mean of MFI of Tc-GFP positive cells in Tc infection (Tc), and astrocytes infected by HIV and superinfected by Tc (HIV/Tc). **(E)** Measurement of variation in the Tc DNA level (as fold change) by qPCR in astrocytes only infected by Tc (Tc), and in astrocytes infected by HIV at first and superinfected by Tc (HIV/Tc). **(F)** Measurement by flow cytometry of HIV (black), Tc (dark gray), and HIV + Tc (light gray)-infected astrocytes by determining the frequencies of HIV-GFP+, Tc-DsRED+, and HIV-GFP+/Tc-DsRED+ cells. **(G)** A representative dot plot diagram obtained by flow cytometry during Tc and/or HIV infection quantification of cultured astrocytes is shown. **(H)** Fluorescence microscopy showing Tc (Red: K98-Alexa fluor 647-labeled intracellular amastigotes) and/or HIV (Green: p24 FITC-labeled) infected-primary human astrocytes. Cell and parasite nucleus were stained by DAPI (blue). Graphics are showing values obtained from three independent experiments. Data are given as the mean ± SD Significant differences are indicated by ^*^p < 0.05, ^**^p < 0.01, and ^***^p < 0.001, respectively.

We and others have demonstrated that astrocytes can be productively infected by HIV (Barat et al., 2018; Ojeda et al., 2018; Li et al., 2020) and when *T. cruzi* is concomitantly present, both pathogens may interact (Urquiza et al., 2017). To assess the influence of HIV infection of astrocytes on its posterior *T. cruzi* superinfection and multiplication, primary cultured human astrocytes were exposed subsequently to both pathogens (Figure 3A). The parasite infection and multiplication were evaluated 24 h after *T. cruzi* exposure. The rate of parasitism was increased significantly when astrocytes were previously exposed to HIV in comparison with those non-exposed to HIV as shown in Figure 3B. Also, the multiplication of *T. cruzi* in astrocytes was also significantly enhanced (Figures 3C,D). HIV enhancement of parasite multiplication was also detected by quantifying amastigotes microsatellite DNA sequence (TCZ) using real-time PCR (Figure 3E). Furthermore, using both flow cytometry analysis and immunofluorescence microscopy, we coincidentally observed that after challenging astrocytes with both pathogens, monoinfected cells were the most abundant, while the HIV/*T. cruzi* coinfected astrocytes were found to a lesser extent (Figures 3F–H). Taken together, these observations pointed out that a previous HIV infection of astrocytes increases their subsequent infection by *T. cruzi* as well as parasite multiplication, without implying the intracellular cohabitation of both pathogens as *sine-qua-non* condition.

### Intracellular HIV-Induced ROS Contributes to Increasing *T. cruzi* Infection and Multiplication in Astrocytes

**Figure 4:**
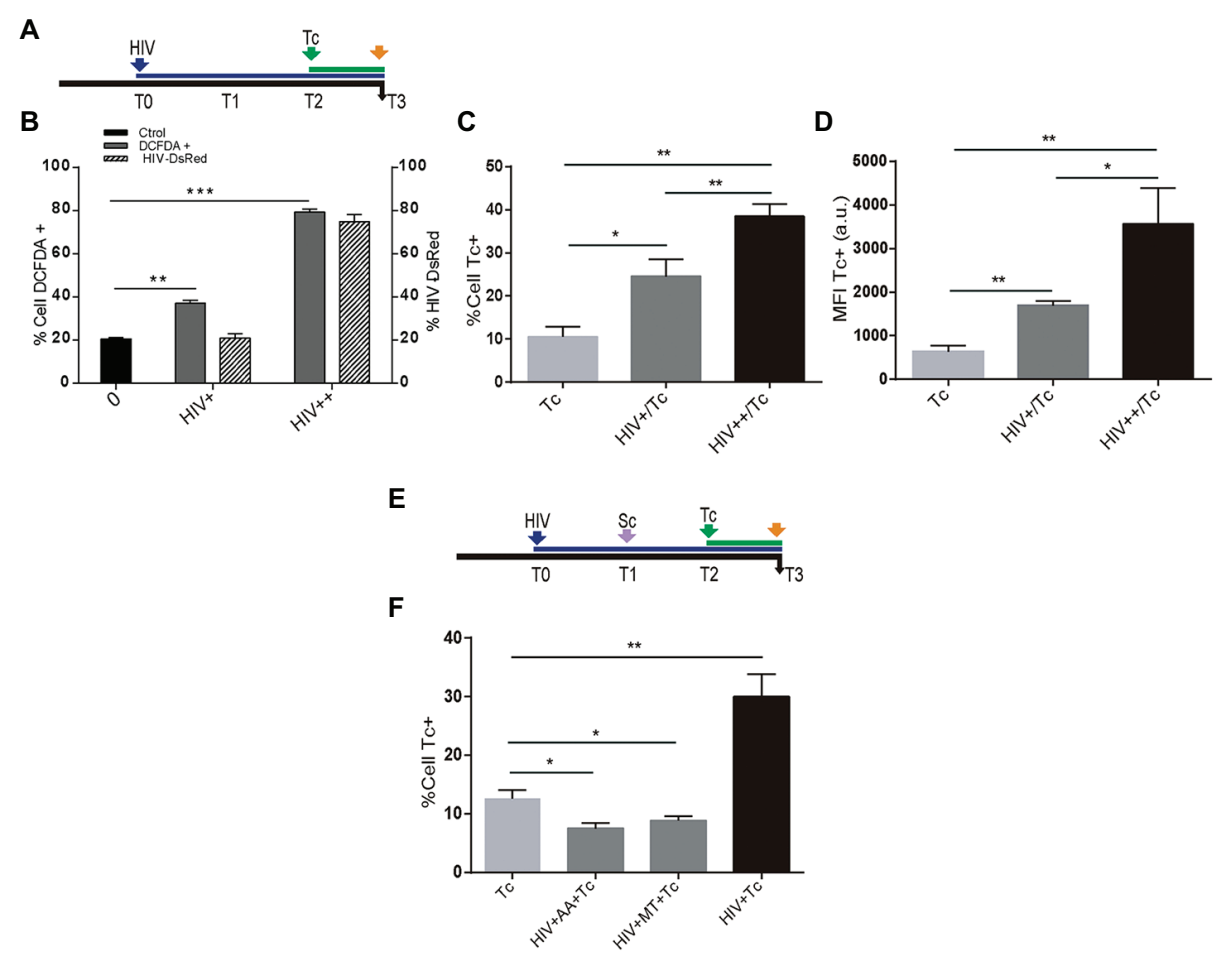
Role of HIV-induced ROS on *Trypanosoma cruzi* infection and multiplication in astrocytes. **(A)** Timeline of the HIV exposure (blue arrow) of cultured astrocytes followed by Tc infection (green arrow, 48 h after HIV exposure). Parameters were measured at T3 (orange arrow, 24 h after Tc exposure). **(B)** Cellular ROS levels (as a percentage of DCFDA-positive cells, on left y-axis) and HIV infection efficiency (measured by flow cytometry as a percentage of GFP-positive cells, on the right y-axis) using two different inoculums at T0 (+: 8 μg/ml, ++: 80 μg/ml of p24 antigen). **(C)** Tc-infection rate measured by flow cytometry analysis in HIV non-exposed (Tc) and HIV-exposed cells (at the two inoculums indicated as HIV+/Tc and HIV++/Tc). **(D)** Tc-multiplication quantification by flow cytometry as mean fluorescence intensity (MFI, expressed in arbitrary units) in HIV non-exposed (Tc) and HIV-exposed astrocytes (at the two inoculums indicated as HIV+/Tc and HIV++/Tc). **(E)** Timeline of the HIV exposure (blue arrow) of cultured astrocytes followed by scavenging (MT: MitoTEMPO; AA: ascorbic acid; violet arrow, 24 h after HIV exposure), and Tc infection (green arrow, 48 h after HIV exposure). Parameters were measured at T3 (orange arrow, 24 h after Tc exposure). **(F)** Tc-infection rate measured by flow cytometry analysis in HIV non-exposed astrocytes followed by Tc infection (Tc) and in HIV-exposed cells (at the two inoculums as indicated), then scavenged (with MT, or AA), and finally exposed to Tc. A control without pretreatment with scavengers is included (HIV + Tc). Graphics are showing values obtained from three independent experiments. Data are given as the mean ± SD Significant differences are indicated by ^*^p < 0.05, ^**^p < 0.01, and ^***^p < 0.001, respectively.

Recently, we have reported that HIV infection increases ROS generation in astrocytes (Ojeda et al., 2018). Resembling the HIV-mediated enhancement of *T. cruzi* infection described above, we were prompted to study whether HIV-induced ROS production on astrocytes is a dose-dependent phenomenon that may influence susceptibility to *T. cruzi* infection and multiplication (Figure 4A). For this goal, astrocytes were challenged with two inoculums of HIV: 8 and 80 μg/ml, thus increasing both the rate of HIV infection and the astrocyte ROS level (Figure 4B). After the challenge with *T. cruzi*, these astrocytes exhibiting significantly increased *T. cruzi* infection and multiplication (Figures 4C,D). When HIV infection of astrocytes was followed by scavenger treatment before *T. cruzi* exposure (Figure 4E), the level of parasitism was even significantly lower than untreated cells (Figure 4F).

Taken together, these results demonstrate that infection and multiplication of *T. cruzi* in astrocytes increase when cells are previously exposed to HIV, since this raises ROS activity.

## Discussion

In immunosuppression conditions, the reactivation of *T. cruzi* infection occurs mainly in the CNS. In this context, neurological involvement is observed is the vast majority of HIV-coinfected patients (Ferreira et al., 1997; Cordova et al., 2008). Astrocytes have been proposed as parasite (Weinkauf et al., 2011; Silva et al., 2015, 2017), and also HIV target cells, as we and others have reported (Urquiza et al., 2017; Chen et al., 2020; Li et al., 2020; Proust et al., 2020).

The ROS are paradigmatically assumed as antimicrobial defense weapons exhibited by phagocytes. It can be generated using both enzymatically and non enzymatically processes being those that occur into the mitochondria one of the main contributors (Nathan et al., 1979). In the CNS, in addition to microglial cells, the activated astrocytes are also source of ROS production (Sharma et al., 2007; Williams et al., 2010; Sheng et al., 2013). Under normal conditions, the ROS produced can be maintained in homeostasis without triggering deleterious effects to the cell host by the coordinated action of antioxidant enzymes and molecules. However, under inflammation among other pathological scenarios, ROS can damage cells (Sheng et al., 2013).

Trypomastigotes can actively infect a variety of non-immune and immune cells and also may be phagocytosed by macrophages (Walker et al., 2014). Such event activates a rapid increased in the production of inflammatory cytokines (Koo et al., 2018), and concomitantly, inducible NADPH oxidase (NOX2) produces superoxide that can be transformed suddenly or enzymatically by superoxide dismutases to H_2_O_2_, which is also dismutated by cytosolic glutathione peroxidase and peroxidase (Gupta et al., 2011). Likewise, inducible nitric oxide synthetase (iNOS) produces NO that can react with superoxide and generate peroxynitrite in infected macrophages. Therefore, *T. cruzi* is regularly exposed to ROS throughout its life cycle and needs to effectively manage the antioxidant and reparation systems to overwhelm the toxic effects of oxidative stress. To this goal, this parasite expresses antioxidant enzymes that are crucial to defending against oxidative damage, allowing the parasite to surpass such oxidative conditions (Paiva et al., 2012; Goes et al., 2016; Hugo et al., 2017). Nevertheless, *T. cruzi* produces its own metabolism-derived ROS, and during the intracellular stages of the lifecycle, it must deal with ROS from the host cell oxidative stress. Thus, for its survival *T. cruzi* has developed ROS detoxification and DNA repair pathways (Mesias et al., 2019). Moreover, recent evidence indicates that the amastigotes division in macrophages and cardiomyocytes is stimulated in ROS enriched oxidative environments by increasing iron availability from host’s cellular reservoir (Gupta et al., 2009; Paiva et al., 2012, 2018; Goes et al., 2016; Dias et al., 2017). Almost all cells need iron as an essential micronutrient, operating as a cofactor for multiple metabolic enzymes. *T. cruzi* demands a large quantity of iron for growth and differentiation (Dick et al., 2020). When macrophages are exposed antioxidants, then the availability of iron decreased and provokes a higher expression of ferritin (a protein that binds iron) and ferroportin-1 (a channel for iron efflux). A low iron level correlates with a lower parasite cargo. Consequently, the use of an iron chelator such as desferrioxamine has been reported to be useful for reducing parasitemia and mortality in experimentally infected mice (Arantes et al., 2007). Besides, the HIV replication maybe also influenced when the iron is accumulated or depleted, free radical synthesis is promoted, and inflammation and mitophagy is enhanced (Allen et al., 2013; Andersen et al., 2014). In the CNS, it was insinuated that an HIV-associated dysregulation of iron transport in the brain may occur, including the possibility of iron deficiency in neurons and iron overload in astrocytes (Mehta et al., 2017). However, it is still unknown the iron role on *T. cruzi* life cycle in astrocytes. Thus, mechanisms by which ROS promote *T. cruzi* infection are still to be fully explained. Current speculation sustains that two possible evolutionary and interacting pressures that forced the selection of *T. cruzi* proliferative response are the increased availability of micronutrients (such as labile iron) and the decreased activation of efficient microbicide mechanisms under oxidative stress (Paiva et al., 2018). Several research groups have explored the possibility that the oxidative environment is itself a direct stimulus to the growth of *T. cruzi*. As an example, trypomastigotes responded to incubation with H_2_O_2_ before the infection, giving rise to greater amastigote cargo after they invaded macrophages or fibroblasts (Aguiar et al., 2013). Likewise, cruzipain is a parasite enzyme that increases the susceptibility of macrophages to parasite infection (Stempin et al., 2008) and is a major inducer of NOX2 activation during macrophage infection (Guinazu et al., 2010).

Astrocytes are the most abundant in a location, where the parasite frequently reactivates in immunocompromised patients, as those with AIDS. Here, we demonstrate that ROS produced by astrocyte during parasite infection also fuels significatively its infection and multiplication. Likewise, as the production of mitochondrial ROS can be potentiated by exogenous oxidants (Jou, 2008), we have used an exogenous source of peroxide (TBH) to promote such enhancement. We observed a pronounced augment in the levels of infection and multiplication of the parasite in a concentration-dependent manner. Thus, with the highest concentrations of the pro-oxidant agent, a significant accumulation of amastigotes was observed that may correlate with the increased astrocyte death observed, as occurs among cardiomyocytes at later stages of parasite multiplication (de Souza et al., 2010; Manque et al., 2011). Furthermore, such ROS-dependent exacerbation of the *T. cruzi* infection and multiplication in astrocytes was counteracted when mitochondrial and cellular ROS were scavenged. Likewise, similar findings were reported in macrophages. The incubation of infected macrophages with up to 100 μM H_2_O_2_ promotes a more intensive amastigote proliferation, but the concentrations of H_2_O_2_ that reach the cytosol are unknown. During the macrophage respiratory burst, the cytosolic concentration of H_2_O_2_ would be around 1–4 μM (Paiva et al., 2012). We have observed that astrocytes viability at concentrations of TBH higher than 250 μM was lower than 10% (data not shown). Nevertheless, a concentration of H_2_O_2_ as high as 300 μM is unlikely to occur *in vivo* (Huang and Sikes, 2014).

Remarkably, other cell types present in the intact CNS could contribute to the regulation of oxidative state experienced by astrocytes. Hence, the progression of HIV infection toward more advanced stages is accompanied by iron accumulation on macrophages and, at the CNS, microglia. The iron excess may enhance the oxidative stress which impairs immune mechanisms. As well, microglial cells are also susceptible to be infected by this parasite, thus generating another cellular scenario for cohabitation. However, astrocytes appear to be more permissive to parasite replication because the activation of the NLRP3 inflammasomes is lower than in microglia-infected cells (Pacheco et al., 2019).

Astrocytes are host-cells for both pathogens (Blanchet et al., 2010; Vargas-Zambrano et al., 2013; Urquiza et al., 2017). Then, in a coinfection – with cellular cohabitation or by bystander effect between monoinfected cells – the progress of the infections can be modified. Such cell-to-cell interactions may involve the participation of EVs, as was reported for *T. cruzi* parasitized cells (Goncalves et al., 1991; Trocoli Torrecilhas et al., 2009). The interaction between *T. cruzi* released EVs and target cells may modulate the host responses against the parasite (Cestari et al., 2012; Ramirez et al., 2017). Similarly, EVs obtained from *T. cruzi* tissue-culture cell-derived trypomastigotes promote functional changes in host-target cells that enhance its infection (Retana Moreira et al., 2019), as well as to modulate the host immune response in favor of the parasite and carry different virulence factors (Ramirez et al., 2017; Caeiro et al., 2018; Lovo-Martins et al., 2018). In line with these reports, we were able to demonstrate that EVs from free trypomastigotes, but not from intracellular amastigotes, were able to increase the host cellular and mitochondrial ROS level which consequently enhanced *T. cruzi* infection in astrocytes, in a concentration-dependent manner. Therefore, this EVs released by the parasite can contain enzymes that increase the ROS production after inducing cell host NADPH oxidase activity (Guinazu et al., 2010), thus increasing the susceptibility to infection (Stempin et al., 2008). Moreover, under pro-oxidant conditions, EVs shedding is induced (Benedikter et al., 2018), which also may carry different antioxidant enzymes involved in ROS scavenging (Bodega et al., 2019).

We have previously reported that HIV infection increases intracellular ROS levels in astrocytes. Such oxidative stress was observed among productively and non-productively infected cells, as a bystander effect. Nevertheless, the astrocytes productively infected with HIV but not the non-productively infected ones were able to mitigate ROS production. So, intracellular ROS concentration remained high among non-productive HIV infected cells (Ojeda et al., 2018) as a consequence of its diffusion through channels in the plasma membrane, or promoted by soluble HIV-released proteins released from infected cells (Song et al., 2007). This cellular scenario allows explaining the very low frequency of astrocytes with intracellular coexistence of both pathogens observed in the present study, emphasizing that the synergic interplay between both pathogens may dispense with the intracellular cohabitation.

Currently, the genotypes of *T. cruzi* are assembled in seven discrete typing units (Jansen et al., 2020). It was documented an increased production of ROS and the resulting oxidative damage during both acute and chronic stages of *T. cruzi* infection with strains from different DTUs (Wen et al., 2010, 2014; Dhiman and Garg, 2014). Then, a higher level of parasites was found in the blood, skeletal muscles, and hearts of mice treated with a NADPH oxidase 2 (NOX2) inhibitor during the acute infection with clone Sylvio (DTU-I; Dhiman and Garg, 2011). The opposite was observed in NOX2-deficient mice infected with either Y strain (DTU-II) or clone CL-Brener (DTU-VI), showing a decreased parasite burden in the peritoneal and splenic macrophages (Paiva et al., 2012; Goes et al., 2016). Further comparative studies are deserved considering that it is still unknown whether such differences are related to *T. cruzi* DTU type involved or, to the distinct functions of the ROS enzymes.

**Figure 5:**
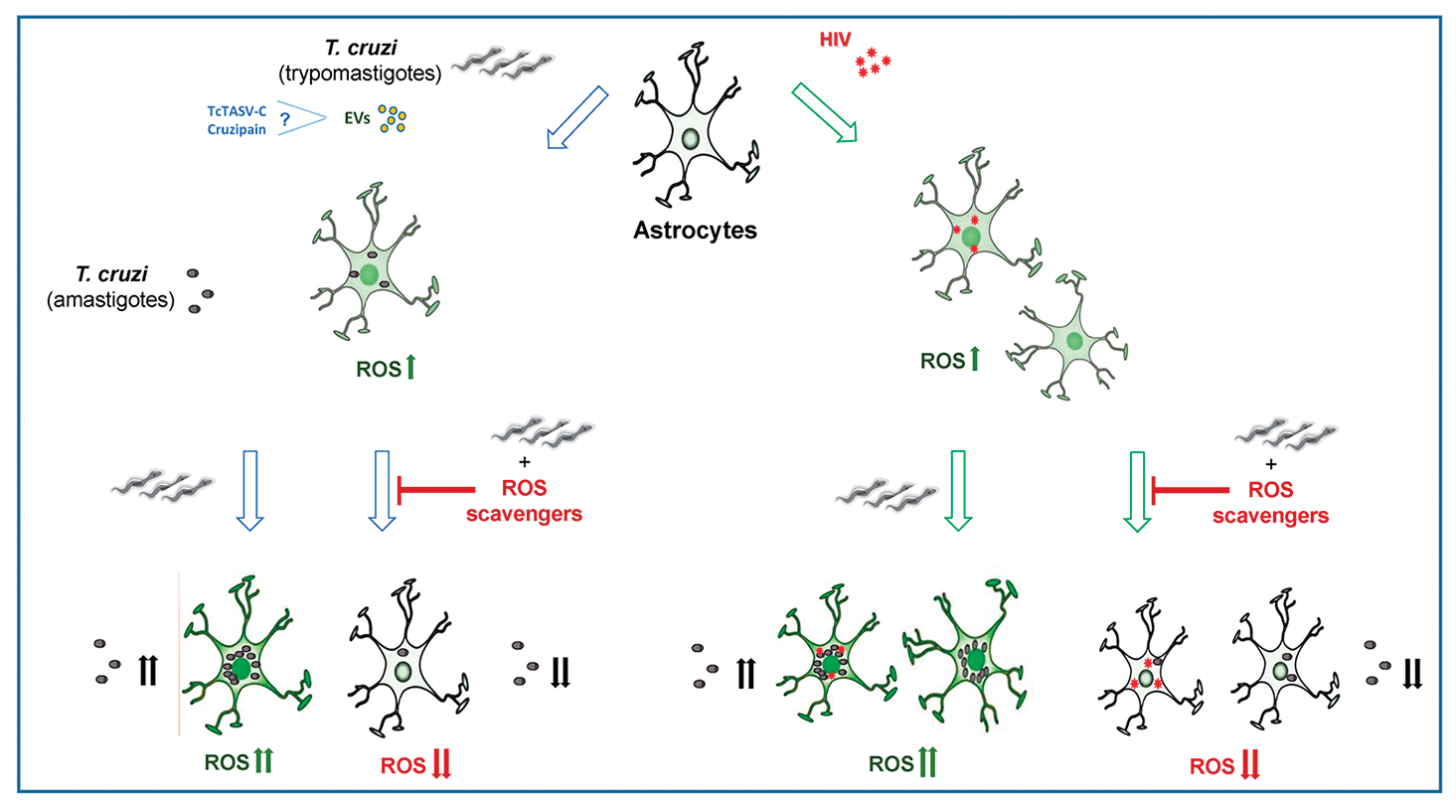
The infection and multiplication of *Trypanosoma cruzi* in astrocytes is influenced by reactive oxygen species (ROS) generated during its infection or by prior HIV exposure.

In conclusion, the self-sustaining ROS drives astrocyte infection by *T. cruzi*, and involve ROS production from HIV-exposed astrocytes during coinfection, contributing to parasite persistence and CNS pathology. Our original insights shed light on the pathogenesis of the neurologic Chagas disease, offering deeper information that supports the design of new parasite control strategies (Figure 5).

## Data Availability Statement

The raw data supporting the conclusions of this article will be made available by the authors, without undue reservation.

## Author Contributions

JU, CC, and ME performed the experiments. All authors analyzed the data. JU, MD, and JQ wrote the article. MD and JQ designed the experiments, revised the article, and obtained research funding. All authors have read and approved the final manuscript.

### Conflict of Interest

The authors declare that the research was conducted in the absence of any commercial or financial relationships that could be construed as a potential conflict of interest.
